# Integral Dose-Based Inverse Optimization May Reduce Side Effects in Radiotherapy of Prostate Carcinoma

**DOI:** 10.3389/fonc.2017.00027

**Published:** 2017-03-01

**Authors:** Ivaylo B. Mihaylov

**Affiliations:** ^1^Department of Radiation Oncology, University of Miami, Miami, FL, USA

**Keywords:** dose, mass, quantitative, imaging, IMRT, energy, prostate, optimization

## Abstract

**Purpose:**

The purpose of this work is to apply a novel inverse optimization approach, based on utilization of quantitative imaging information in the optimization function, to prostate carcinoma.

**Materials and methods:**

This new inverse optimization algorithm relies upon quantitative information derived from computed tomography (CT) imaging studies. The Hounsfield numbers of the CT voxels are converted to physical density, which in turn is used to calculate voxel mass and the corresponding integral dose, by summation over the product of dose and mass in each dose voxel. This integral dose is used for plan optimization through its global minimization. The optimization results are compared to the optimization results derived from most commonly used dose–volume-based inverse optimization, where objective functions are formed as summation over all dose voxels of the squared differences between voxel doses and user specified doses. The data from 25 prostate plans were optimized with dose–volume histogram (DVH) and integral dose (energy) minimization objective functions. The results obtained with the energy- and DVH-based optimization schemes were studied through commonly used dosimetric indices (DIs). Statistical equivalence tests were further performed to establish population-based significance results.

**Results:**

Both DVH- and energy-based plans for each case were normalized so that 95% of the planning target volume receives the prescription dose. The average differences for the rectum and bladder DIs ranged from 1.6 to 25%, where the energy-based quantities were lower. For both femoral heads, the energy-based optimization-derived doses were lower on average by 32%. The statistical tests demonstrated that the significant differences in the tallied dose indices range from 2.7% to more than 50% for rectum, bladder, and femoral heads.

**Conclusion:**

For majority of the clinically relevant dosimetric quantities, energy-based inverse optimization performs better than the standard of care DVH-based optimization in prostate carcinoma. The population averaged statistically significant differences range from ~3 to ~50%. Therefore, this newly proposed optimization approach, incorporating explicitly quantitative imaging information in the inverse optimization function, holds potential for further reduction of complication rates in prostate cancer.

## Introduction

Radical prostatectomy and radiotherapy are both commonly used treatment options for clinically localized prostate cancer (1). Both treatment approaches have evolved over time, such that to maximize cancer-free survival, while minimizing health-related risks like urinary incontinence, bowel incontinence, and sexual dysfunction. However, to date, there is still no superior single treatment that is devoid of side effects (1). Three recent randomized trials have shown a consistent improvement in biochemical failure when adjuvant radiotherapy is administered with prostatectomy (2). However, the price to be paid for the increased control is the increased risk for complications and side effects in the radiotherapy arm by a factor of 2 (3).

Over the past decade, many publications have shown that high dose intensity-modulated radiotherapy (IMRT) represents optimal form of external beam radiotherapy for localized prostate cancer (4). The combination of IMRT with image guidance (5) has allowed an enhanced target precision, together with the accompanying reduction in clinical target volumes (CTVs) and even perhaps reduction in the treatment-related toxicity. Those findings suggest that the development and the incorporation into clinical practices of novel advanced radiotherapy planning and delivery techniques will allow safer radiation delivery for numerous of patients with potentially lower side effects.

Modern radiotherapy treatment planning relies on the dose–volume histogram (DVH) paradigm, where doses to volumes are employed (6–9). The widespread use of DVHs stems from the wealth of clinical information as well as clinician’s experience with dose–volume metric (10). DVHs were introduced three decades ago, while IMRT gained widespread application a decade later (9, 11–15). At that time, homogeneous dose calculations were the norm, with heterogeneous dose calculations hardly even possible. In the last two decades, however, these shortcomings were overcome, and today, heterogeneous dose calculations are the norm in all commercial treatment planning systems (TPSs). Despite the use of more sophisticated dose calculation algorithms, however, the inverse IMRT optimization is still based to a large extent on the DVH paradigm which, by its virtue, is homogeneous in nature. In other words, all voxels in the irradiated volume are treated by the optimization cost function derived only in terms of doses and volumes, thereby ignoring the fact that the voxels have inhomogeneous content. Notably, the computed tomography (CT) imaging data provide voxel density information which can be used beyond its application to radiation attenuation and absorption (dose deposition). This fact has been pointed out in a recent study where novel optimization cost function was introduced (16). In this work, the inhomogeneity of the voxels was directly incorporated into the cost function, where the voxel mass utilization was achieved through integral dose (energy-based optimization hereafter).

The purpose of the present work is to investigate the applicability of this novel energy-based optimization scheme to prostate cancer. The abdominal region consists of almost uniform density, where the dose calculation accuracy will minimally impact the obtained optimization solution (17). Thereby, if there are any observable differences between contemporary inverse optimization techniques and this newly proposed form of the cost function, they will result from the intrinsic properties of this cost function itself.

## Materials and Methods

### Patient Data

This study was carried out in accordance with the recommendations of University of Miami Institutional Review Board (IRB) guidelines, with written informed consent from all subjects. All subjects gave written informed consent, in accordance with the Declaration of Helsinki, as per University of Miami IRB approved protocol for this study. The CT data from 25 prostate cancer subjects were used in this study. For each case, CTV, including only the prostate, and planning target volume (PTV) were contoured by the attending physician. The prescription doses ranged from 70 to 80 Gy in 2 Gy daily fractions. The PTV was obtained from the CTV by a uniform expansion of ~1 cm. In addition, rectum, bladder, and femoral heads were outlined as organs at risk (OARs) and were used in the IMRT optimization as dose limiting structures.

### Objective Functions

The objective functions used in the inverse optimization of the treatment plans are described below. They are strictly limited to the OAR doses, while the target dose objectives were specified in terms of minimum, maximum, and uniform doses to the PTV. The DVH-based optimization is realized through an objective function described by Eq. 1

(1) $$F^{j}=\sum_{i\in V,d_{i}>d^{j}}{\left(\frac{d_{i}-d^{j}}{d^{j}}\right)}^{2}\frac{\Delta v_{i}}{V}\text{,}$$

where *V* denotes the volume of interest (VOI), *d_i_* is the dose in voxel (3D volume element) *i* of the volume *V, d^j^* is the desired dose in each voxel, and Δ*v_i_* is the voxel volume (16, 18, 19). The summation is over the volume for which the dose *d_i_* is greater than the objective dose *d^j^*. This equation can also be applied for minimum, maximum, and uniform dose objectives, where the summations are over the volumes with *d_i_* smaller than *d^j^, d_i_* greater than *d^j^*, and the entire organ volume, respectively. An optimization function *F^j^* is created for each optimization objective specified for the organ of interest. In the case of DVH-based optimization, more than one objective can be specified for an anatomical structure of interest. A summation over all objective functions *F^j^* generates a composite objective function outlined in Eq. 2

(2) $$F=\sum_{j=1}^{N}F^{j}\text{.}$$

The normalization of the voxel volume *v_i_* with respect to the total volume *V* of the anatomical structure of interest in Eq. 1 is needed so that the composite objective function of Eq. 2 can be generated. This normalization assures that all of the individual OAR *F^j^* are dimensionless, and they can be combined with the objective functions for the target(s) (which are also dimensionless), thereby creating the global objective function from Eq. 2.

So far integral dose has only been used to explore the properties of treatment plans (20–22). However, it has been argued recently that integral dose has some far reaching desirable properties, which can be used in radiotherapy inverse plan optimization (16). The integral dose represents the total energy deposited in a VOI. Therefore, minimizing the integral dose is in essence a minimization of the total deposited energy in that VOI. The discrete form of the objective function *F^j^* used in this work is presented in Eq. 3

(3) $$F^{j}=\frac{1}{E_{\text{objective}}}\sum_{i\in V}d_{i}\rho_{i}v_{i}=\frac{1}{E_{\text{objective}}}\sum_{i\in V}d_{i}m_{i}\text{,}$$

where again *V* denotes the volume of the anatomical structure of interest, *d_i_* is the dose in voxel *i*, ρ*_i_* is the density of the material in that voxel, *m_i_* is the mass of that material, and *v_i_* is the volume of the voxel (16). The summation is over all dose voxels contained in the volume of the organ of interest *V*. The physical density ρ in each voxel is obtained from the Hounsfield units (HUs) contained in the DICOM CT images. The conversion of the HUs to physical density is performed though CT-to-density conversion table, which is present in each TPS since it is necessary for the dose calculations. The quantity *E*_objective_ in Eq. 3 is the desired integral dose, which needs to be imparted on the organ of interest. This in essence is a normalization term, which ensures that the individual objective functions *F^j^* are dimensionless, in analogy to the DVH-optimization case (cf. Eq. 1). The normalization allows the combination of dimensionless OAR objectives with the dimensionless objective functions for the target(s) in Eq. 2. For each anatomical structure considered in the optimization, there is only one energy-based objective function of the type given by Eq. 3.

Both Eqs 1 and 3 have one adjustable parameter each. While in Eq. 1, it is the desired dose *d^j^*, in Eq. 3, it is the desired integral dose *E*_objective_. The existence of each of those adjustable parameters is a prerequisite for the inverse optimization. By planner’s action on those parameters during the optimization process, the computer optimization algorithm is invoked to change voxel doses *d_i_* (cf. Eqs 1 and 3) during optimization, therefore, causing the solution to converge to a point where the global objective function from Eq. 2 is minimized (i.e., maximum target doses and minimum OAR doses).

### Treatment Planning

**Figure 1:**
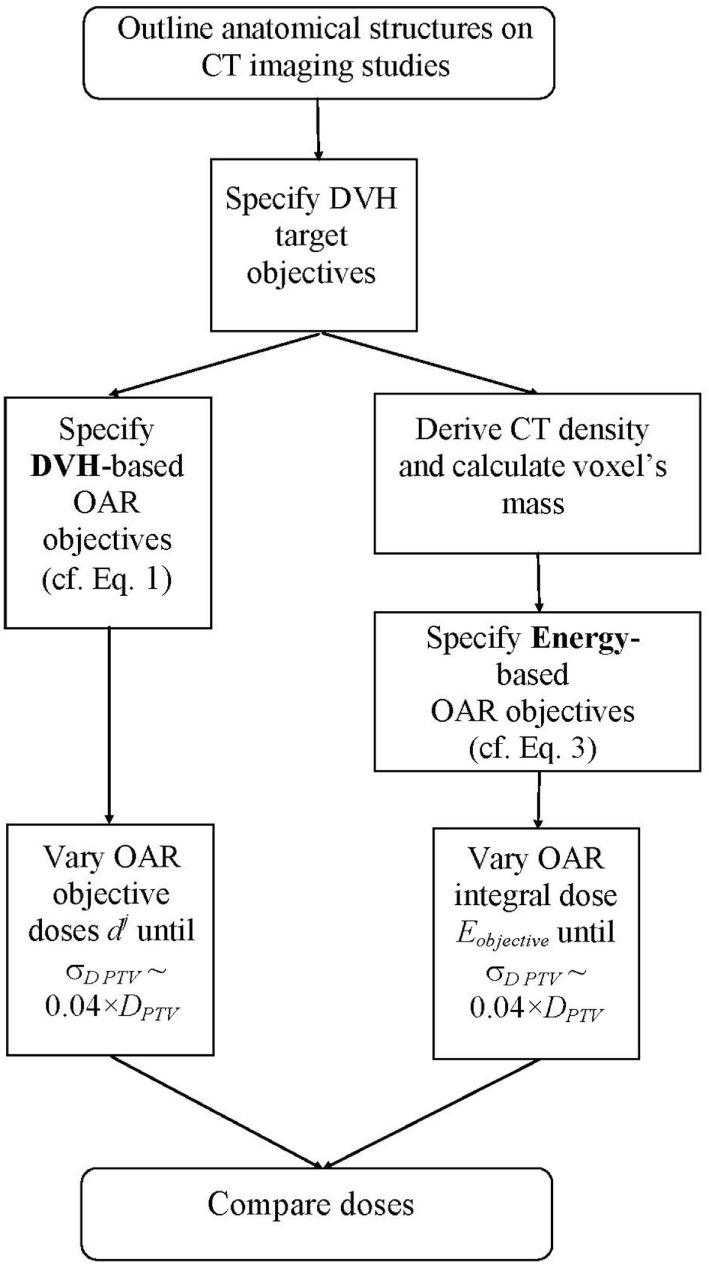
**Flowchart of the optimization process**. The left-hand arm represents the DVH-based optimization sequence, while the right-hand arm outlines the energy-based optimization sequence.

For each patient, two IMRT plans were created: one based on DVH quadratic objective function (9, 18, 19), and another one based on energy-minimization objective function (16). For the PTV doses, both optimization schemes utilized pure dose objective functions, namely, minimum, maximum, and uniform doses. For the OARs, the IMRT objectives were DVH based and energy based in the two arms, respectively. Figure 1 outlines a schematic representation of the study flow. In essence, this is a multistage trail-and-error optimization scheme. With either optimization scheme, there is a full optimization cycle performed with only DVH target objectives. After convergence of the optimization, DVHs for all OARs are created. From those DVHs, the DVH-optimization functions (cf. Eq. 1) are evaluated. There are three objectives per OAR for prespecified relative volumes of 1, 35, and 70% of the OAR. Objective doses *d^j^* are varied such that the individual objectives *F^j^* for all OARs are about 5% larger than the largest objective value, obtained from the target objective functions (usually this is the objective value derived from the target dose uniformity objective function). After the objective doses for the OARs are determined, the entire optimization is performed again. At the end of this optimization cycle, the doses (and, respectively, the DVHs) of the OARs are reduced, while the target dose heterogeneity is increased. The OAR objective doses *d^j^* are evaluated again as described above, namely, until the *F^j^* for all OARs are about 5% larger than the target objective value for dose homogeneity, and the optimization is performed again. Therefore, with each adjustment (reduction) of OAR objective doses *d^j^*, the dose heterogeneity of the PTV dose increases. The process of stepwise reduction of OAR objective doses is terminated when at the end of the optimization run the SD of the doses across the PTV is of the order of 4% of the prescription dose. The obtained solution is thereby multistaged trial and error procedure where OAR objective doses are lowered through multiple optimization runs. The procedure with the integral dose-based optimization is identical, with the only difference that there is only one objective per OAR, and the variable parameters are not the individual doses *d^j^* but rather the *E*_objective_ from Eq. 3.

All IMRT plans were generated with deliverable IMRT optimization, where the final optimization solution incorporated all machine parameters (17, 23–25). The deliverable optimization is more restrictive than the intensity map (only) optimization, but the advantage is that the obtained solution is clinically viable and can be delivered. Both plans for each patient consisted of seven coplanar 6 MV beams. A total of 70 step-and-shoot IMRT segments were allowed in each plan with minimum segment area of 5 cm^2^ and minimum of 5 monitor units (MUs) per segment. If the segment area or the minimum allowed MUs are decreased, then the deliverable optimization may result in overmodulation where quality assurance checks may deem the achieved plans clinically unachievable. Thereby, by restricting the degree of intensity modulation plan deliverability is secured. The investigated optimization cost functions were interfaced to Pinnacle TPS (Philips Radiation Oncology Solutions, Fitchburg, WI, USA) by plugins. This allowed the use of Pinnacle’s dose calculation algorithms as well as its direct machine parameter optimization module in the generation of the deliverable plans. For each patient, the DVH- and the energy-based plans were normalized such that 95% of the PTV received the prescription dose. The OAR doses were optimized until SD of the dose across the PTV in each plan became of the order of 4.0% (26).

### Analyses

DVH optimization is for a large part the standard of care in modern radiation therapy. Therefore, the doses derived from the DVH optimization were used as a reference standard with respect to which the energy-based optimization results were evaluated. The performance of each optimization scheme was assessed on the basis of commonly used dosimetric indices (DIs) (17, 27, 28), since DVH metric is the most commonly used tool in the clinical decision making. The tallied DIs were dose to 95% of the PTV volume, doses to 15, 25, 40, and 60% of rectum and bladder volumes, as well as doses to 10% of the volumes of each femoral head. Furthermore, maximum PTV doses and dose to 5% of the PTV volume were also interrogated.

Statistical equivalence tests were used to determine the minimum dose interval, such that the reference and the compared quantities were deemed statistically equivalent. The tests were performed for each tallied index using two-tailed paired *t*-tests for *p*-values of less than 5% (17, 29). The procedure for the statistical tests is as follows. Absolute doses for each DI were extracted for each optimization scheme. Next, dose equivalency interval was initially set to zero and the *t-* and *p-*values computed for the observed absolute dosimetric differences between the two optimization schemes for each DI. Subsequently, the dose interval was progressively increased in 1 cGy steps until equivalence between the indices was reached (namely, until *p* became larger than 0.05). In essence, the dose equivalence interval indicates when the two solutions are indistinguishable from statistical point of view. Larger the paired (on patient-by-patient basis) absolute dose differences between the DIs, the larger the equivalence interval, the larger the statistical significance level and *vice versa*.

## Results

**Figure 2:**
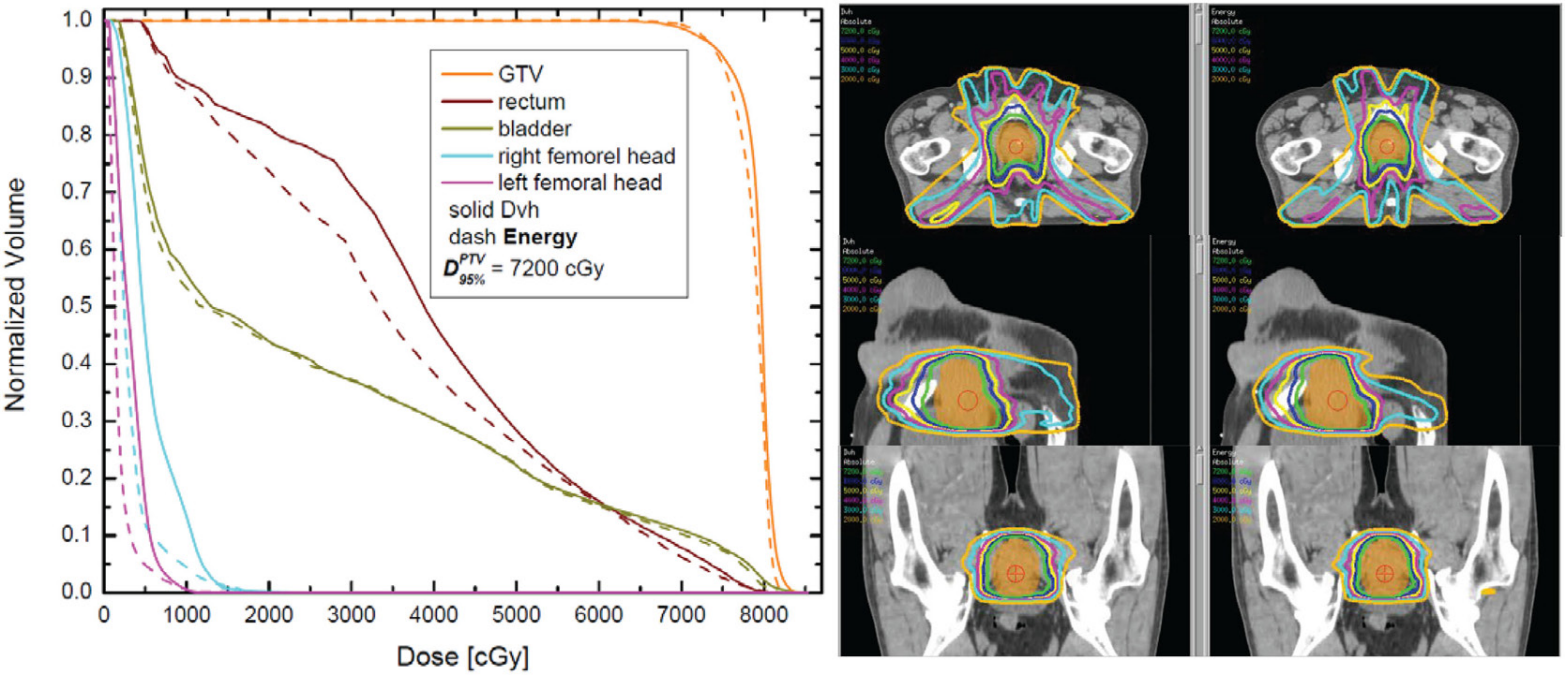
**Dose–volume histogram (DVH) (left) and isodose plots in three different plains (right) for one case**. The DVHs for the planning target volume (PTV), rectum, bladder, and femoral heads are presented on the left. The right-hand side outlines the isodoses from 7,200 cGy (prescription dose) to 2,000 cGy in 1,000 cGy intervals. The axial, the sagittal, and the coronal cuts are through the isocenter, located in the centroid of the PTV.

Figure 2 outlines DVH and isodose comparisons for one case from the cohort. The color-coded DVHs are presented on the left-hand side, while the isodose distributions are presented on the right-hand side. The solid DVHs resulted from the dose distribution derived from the DVH-based optimization, while the dashed DVHs resulted from the energy-based dose distribution. In this particular case, for equivalent PTV coverage (doses are normalized such that 95% of the PTV receives 7,200 cGy with either optimization), the energy-based optimization results in very similar bladder DVH as compared to DVH-based optimization. However, the rectum and the femoral head DVHs are noticeably lower with energy-based inverse optimization. The screen captures on the right-hand side show the DVH-derived (left) and the energy-derived (right) isodoses. As can be noted form these plots, there is somewhat larger “spillage” of low doses (orange, magenta, and cyan) with DVH-based optimization. This difference in the lower dose region is also evident from the DVH overlay on the left-hand side of the figure.

### Comparison of Dose Indices

**Figure 3:**
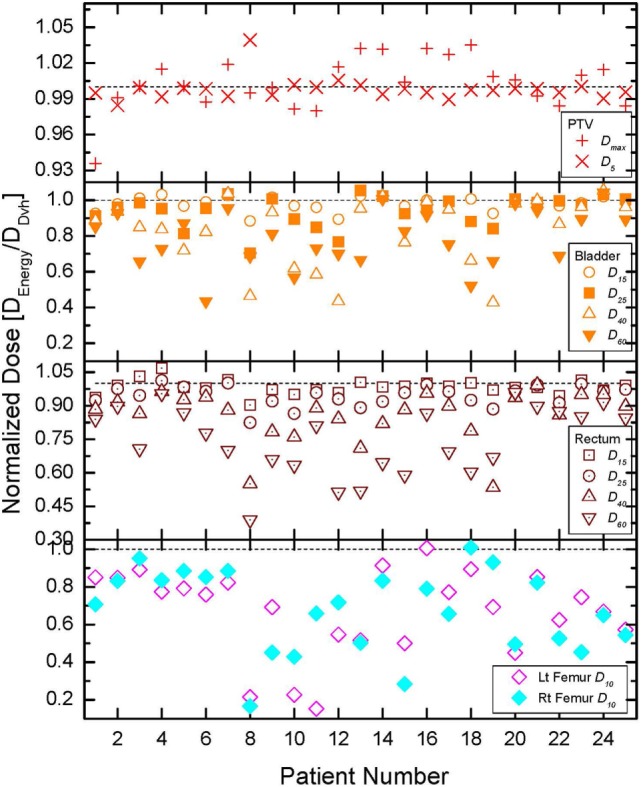
**Comparison of all normalized dose indices for the organs at risk (OARs)**. Details on the index normalization are presented in the text. The top, middle, and bottom panels outline the results for all tallied OAR doses for bladder, rectum, and femoral heads, respectively.

The comparisons of all tallied dose indices are presented in Figure 3. The quantities obtained from the DVH-optimized plans were used as a reference in this normalization, since DVH-based optimization is modern day standard of care. The normalization of the DIs allows the use of a common scale for all patients since the absolute DIs vary from patient to patient due to prescription doses, optimization convergence, and patient anatomy (17, 30). In order to further aid the comparisons, one is plotted on the figure by a dashed line. If a normalized DI is greater than 1 then the DVH optimization results in *lower* absolute value for that quantity and *vice versa*. When a normalized DI is 1, then the DVH- and energy-derived values for that DI are equal. Since the prescription for each patient was performed to 95% of the PTV, those doses were identical to within 1% and are not presented. The top panel of the figure presents the normalized maximum dose and does to 5% of the PTV (see legend). In extreme cases, the differences range from −7% (i.e., less than 1) to +5% (greater than 1), but for the majority of the cases, they are within couple percent of each other. Most of DIs for rectum, bladder, and femoral heads demonstrate that energy-based OAR doses are lower than the DVH-based OAR doses. The top two panels of the figure show that for rectum and bladder, the sparing, achievable with energy-based optimization, increases as the fractional volume of the OAR increases. The average dosimetric differences between the optimization schemes in bladder for instance are 1.9, 5.9, 17.1, and 21.2% (lower energy-derived values) for 15, 25, 40, and 60% respectively of bladder volumes. The trend for the rectum is identical, namely, the differences become larger as the fractional VOI becomes larger. This behavior is to be expected since the goal of either optimization is to spare as much as possible the OARs after adequate target coverage (namely, 95% of the PTV is covered by 100% of the prescription dose) are achieved. Rectum and bladder are in direct proximity to the PTV, and thereby, small fractions of those anatomical structures could receive doses as high as the prescription dose. Therefore, the dose differences for small volumes of those anatomical structures are relatively small. The bottom panel of Figure 3 shows that the sparing of the femoral heads with energy-based optimization is far superior than the sparing with DVH-based optimization. In only 2 out of 50 tallied DIs for the femoral heads, the normalized values are at or above 1 (patients 16 and 18, left and right femoral heads, respectively).

In addition to the commonly used dose indices described above, integral doses were also compared. The entire irradiated volume was delineated. The PTV for each patient was subtracted from this irradiated volume, and the integral doses for each optimization scheme were calculated for each optimization scheme. The normalized (with respect to the DVH-optimization results) integral doses are plotted on the top panel of the figure. As it is clear from the plot, all integral doses for the energy-based optimization are lower, which is in contract to the DIs, where not in all cases the energy-derived DIs were better than the DVH-derived DIs. The integral doses to the irradiated volume were on average lower with energy-based optimization by 8%.

### Statistical Analyses

The results of the statistical significance tests are presented in Table 1. In the first column, a description of the tallied quantity is given. In the second column of the table, the corresponding average value derived from the DVH-optimized plans is presented. For instance, the dose of 6,742 cGy is the average dose to 15% of the bladder volume over all 25 cases, derived from DVH-based plans. Third column contains the interval (in absolute dose units) for which statistical equivalence at *p* < 5% level is achieved. The last column of the table is the ratio between the numbers from third and second columns, therefore, indicating the percent statistically significant difference between the two optimization schemes. Note that in the high dose regions for the rectum and bladder, the statistically significant difference is only about 3%, while with increasing volume, the statistically significant differences increase to about ~30%, which is consistent with the data presented in Figure 3. The statistically significant differences to 10% of the volumes of the femoral heads are in excess of 50%. These findings are exemplified on the DVHs presented in Figure 2. For that particular patient, the bladder doses are not much different, but the rectal doses show how with decreasing dose the DVH resulting from the energy optimization is lower than the DVH derived from the DVH-optimized plan. While the differences in maximum doses and doses to 5% of the PTV range from −7 to +5% (cf. top panel of Figure 3), the statistical equivalence tests indicate that over the patient cohort those differences are significant at ~1% level as indicated by the last two rows in Table 1.

**Table 1:** **Intervals for which statistical equivalence tests indicate that the differences between DVH- and energy-derived dose indices are statistically significant (*p* < 0.05)**.

Index	Average value of tallied index derived from DVH optimization (cGy)	Statistical equivalence interval (cGy)	Change for equivalency (sparing) (%)
Bladder DI_15%_	6,742	185	2.7
Bladder DI_25%_	5,861	377	6.4
Bladder DI_40%_	4,086	696	17.0
Bladder DI_60%_	2,619	692	26.4
Rectum DI_15%_	6,603	198	3.00
Rectum DI_25%_	5,861	402	6.9
Rectum DI_40%_	4,233	792	18.7
Rectum DI_60%_	2,763	953	34.5
Rt femoral head DI_10%_	1,091	553	50.7
Lt femoral head DI_10%_	1,059	579	54.7
Planning target volume DI_5%_	8,297	62	0.7
*D*_max_	8,505	90	1.1

*The average quantities in column two are derived from the standard (DVH-based optimization)*.

## Discussion

Prostate region is considered to be rather homogenous. In reality, however, this is not the case. Density varies by 5 to 10% over the rectum and bladder on average. In the case of the femoral heads, the differences are even more dramatic and can as high as 50%. Therefore, exploiting the density variation through energy-based inverse optimization turns out to be advantageous with respect to dose–volume-based optimization as in the DVH case. It has been shown in prior studies that when different dose calculation algorithms are used the density differences may affect the convergence of the optimization solution where differences close to 10% have been observed (17). Therefore, the use of voxel mass explicitly in the optimization objectives is a logical step in the study and the evolution of the optimization objective functions.

As it was noted above, DVH optimization usually requires several objectives for each OAR, while energy-based optimization utilizes a single objective per OAR. This may be particularly relevant to serial structures where usually maximum doses are of primary concern. The availability of several objective functions for a single OAR (DVH case) allows more flexibility in shaping maximum dose than in the case with only one available objective function (energy case). There are several potential solutions which can be utilized for amelioration of this deficiency of the energy-based approach. One possibility would be to specify an additional (to the energy based) objective for each serial structure where the point maximum dose to the OAR is limited. Alternative option would be to use a DVH-based objective for the OAR, where the dose to a small fraction of the volume (i.e., 1%) is minimized. A third option would be to introduce an additional objective function, which is based on modification of Eq. 3. This modification will minimize integral dose only for voxels that have doses above certain user-defined threshold.

To our knowledge, integral dose has not been related to normal tissue complications. Dose–volume parameters, however, are well known to clinicians because of the vast clinical experience, which has been gathered since the introduction of DVHs (9, 12). Therefore, integral dose cannot solely be used for radiotherapy plan evaluation. Energy-based optimization appears to be a useful alternative for inverse optimization, while the clinical guidelines and clinical trial protocols in terms of established DVH metrics and guidelines should be followed.

Another comparison performed in this investigation was between the MUs, the integral doses, and the surface (skin) doses resulting from each optimization scheme. The average, minimum, and maximum MUs derived from the DVH plans were 752.7, 505, and 1,026, respectively. In comparison, the quantities resulting from the energy-based plans were 760.3, 547, and 1,011 MUs, respectively. The energy-based plans resulted in slightly higher average MUs over the patient cohort, but that difference was small (about 1%). Integral doses within the entire irradiated volume were calculated for each patient and each optimization approach. In all cases without exception, the integral dose derived from the energy-based plan was lower than the integral dose derived from the DVH plans. The differences ranged from 0.1 to 3.1%, with average difference of 1%. As it was mentioned above, there is no evidence in the literature what the effect of those integral dose differences might be. Finally, in order to estimate the surface doses, a 0.3 cm layer was outlined on the patient surface, representing patient’s skin. The maximum and average skin doses were calculated on patient-by-patient basis. The differences in maximum doses ranged from −12.8% (lower skin dose with energy-based optimization) to +12.1% (higher skin dose with energy-based optimization), with average difference of −0.4%. For average skin doses, the minimum, maximum, and average differences were −16, 5.7, and −7%, respectively. These findings indicate that energy-based optimization delivers slightly lower dose to skin. However, only in four cases, the actual skin dose was over 3,000 cGy (and only in two cases around 3,500 cGy). Even in these extreme scenarios, the maximum skin dose is about 100 cGy per day over the course of treatment. The calculations do not account for patient daily repositioning, which will smear out the maximum dose somewhat, thereby decreasing it. Furthermore, if the skin dose is of a concern, the number of beams may be increased, therefore, spreading out the entrance dose, which would decrease the skin dose further.

## Conclusion

The results of this study presented herein indicate that this novel inverse optimization framework, based on the exploration of quantitative imaging information derived from the CT data, is capable of improving normal tissue sparing when compared to the standard of care. The observed statistically significant differences range from about 3% in the high dose regions to more than 50% in the low dose regions. It is interesting to note that this normal tissue sparing is observed in rather homogenous media, characteristic for the prostate population. With DVH-based optimization, the cost function is a quadratic function of dose differences multiplied by volume, while the objective function in energy-based optimization is a product of dose and mass. The observations presented above imply that the minimization of the integral dose allows better normal tissue sparing than DVH-based inverse optimization even in almost homogenous media such as the pelvic region. Since both optimization schemes use the same dose calculation algorithm and the variations in tissue density are relatively small, it can be concluded that the obtained results are property of the underlying optimization cost function. This cost function represents a further evolution of our strive for personalized medicine, where therapy is tailored to specific patient anatomical information, and now tissue density representation. By its virtue, high-energy ionizing radiation destroys human tissue. Thereby, minimizing radiation doses to healthy tissue is one of the primary goals of modern radiotherapy treatment planning and delivery. The main findings in this work strongly suggest that energy minimization-based inverse optimization holds the potential to reduce further toxicity in radiotherapy treatment of one of the most common cancers.

## Author Contributions

The author confirms being the sole contributor of this work and approved it for publication.

## Conflict of Interest Statement

The author declares that the research was conducted in the absence of any commercial or financial relationships that could be construed as a potential conflict of interest.
